# The impact of Medicaid expansion under the Affordable Care Act on HIV care continuum outcomes across the United States

**DOI:** 10.1093/haschl/qxae128

**Published:** 2024-10-07

**Authors:** Peter F Rebeiro, Julia C Thome, Stephen J Gange, Keri N Althoff, Stephen A Berry, Michael A Horberg, Richard D Moore, Michael J Silverberg, Daniel E Sack, Timothy R Sterling, Pedro Sant’Anna, Bryan E Shepherd

**Affiliations:** Department of Medicine, Division of Infectious Diseases, Vanderbilt University School of Medicine, Nashville, TN 37203, United States; Department of Biostatistics, Vanderbilt University School of Medicine, Nashville, TN 37203, United States; Department of Medicine, Division of Epidemiology, Vanderbilt University, School of Medicine, Nashville, TN 37203, United States; Department of Biostatistics, Vanderbilt University School of Medicine, Nashville, TN 37203, United States; Department of Epidemiology, Johns Hopkins Bloomberg School of Public Health, Baltimore, MD 21205, United States; Department of Epidemiology, Johns Hopkins Bloomberg School of Public Health, Baltimore, MD 21205, United States; Department of Medicine, Johns Hopkins University School of Medicine, Baltimore, MD 21205, United States; Mid-Atlantic Permanente Research Institute, Kaiser Permanente Mid-Atlantic States, Rockville, MD 20852, United States; Department of Epidemiology, Johns Hopkins Bloomberg School of Public Health, Baltimore, MD 21205, United States; Department of Medicine, Johns Hopkins University School of Medicine, Baltimore, MD 21205, United States; Kaiser Permanente Northern California, Division of Research, Oakland, CA 94612, United States; Department of Medicine, Division of Epidemiology, Vanderbilt University, School of Medicine, Nashville, TN 37203, United States; Department of Medicine, Division of Infectious Diseases, Vanderbilt University School of Medicine, Nashville, TN 37203, United States; Department of Economics, Vanderbilt University, Nashville, TN 37203, United States; Department of Biostatistics, Vanderbilt University School of Medicine, Nashville, TN 37203, United States

**Keywords:** Medicaid, Patient Protection and Affordable Care Act, HIV, HIV care continuum, clinical retention, ART initiation, viral suppression

## Abstract

HIV care continuum outcome disparities by health insurance status have been noted among people with HIV (PWH). We therefore examined associations between state Medicaid expansion and HIV outcomes in the United States.

Adults (≥18 years) with ≥1 visit in NA-ACCORD clinical cohorts from 2012-2017 contributed person-time annually between first and final visit or death; in each calendar year, clinical retention was ≥2 completed visits > 90 days apart, antiretroviral therapy (ART) receipt was receipt of ≥3 antiretroviral agents, and viral suppression was last measured HIV-1 RNA < 200 copies/mL. CD4 at enrollment was obtained within 6 months of enrollment in cohort. Difference-in-difference (DID) models quantified associations between Medicaid expansion changes (by state of residence) and HIV outcomes.

Across 50 states, 87 290 PWH contributed 325 113 person-years of follow-up. Medicaid expansion had a substantial positive effect on CD4 at enrollment (DID = 93.5, 95% CI: 52.9, 134 cells/mm^3^), a small negative effect on proportions clinically retained (DID = −0.19, 95% CI: −0.037, −0.01), and no effects on ART receipt (DID = 0.001, 95% CI: −0.003, 0.005) or viral suppression (DID = −0.14, 95% CI: −0.34, 0.07).

Medicaid expansion had a positive effect on CD4 at entry, suggesting more timely HIV testing and care linkage, but generally null effects on downstream HIV care continuum measures.

## Introduction

Since its passage in 2010, the Patient Protection and Affordable Care Act (ACA, “Obamacare”) has enhanced and improved health insurance coverage, screening, and health outcomes for under-insured and vulnerable populations across the United States, particularly through opt-in expansions of Medicaid coverage by the states.^1-4^ People with HIV (PWH), particularly before the implementation of the ACA, have been a population of particular vulnerability in the health insurance marketplace. In fact, before the ACA, PWH experienced restricted access to health insurance coverage for multiple reasons, including exclusions due to pre-existing conditions, higher medical costs, and Medicaid eligibility limitations.^5,6^ Central components of the ACA reduced and removed these restrictions. Coverage increased significantly for PWH due to the ACA's Medicaid expansion; indeed, increased Medicaid coverage in expansion states drove a nationwide increase in coverage for PWH, rising from 36% to 42% in the first year of the expansion (2014).^7-9^ In addition, the share of PWH relying on the Ryan White HIV/AIDS Program decreased in expansion states, with as many as 55% transitioning to Medicaid or private insurance.^8,10,11^ Given the fact that the United States may undergo additional changes in federal health insurance standards in the future, examining the impacts of health policy reforms to date on vulnerable populations, such as PWH, is desirable.^12-15^

The HIV care continuum is an instructive framework that has been adopted by public health agencies, policy makers, and epidemiologists to evaluate the engagement of PWH in clinical care, and assess individual health outcomes, HIV disease progression, and HIV transmission. Gaps in the continuum between HIV testing, diagnosis, linkage to care, and retention in care through antiretroviral therapy (ART) receipt and viral suppression represent barriers to successful HIV treatment and potential points of intervention;^16,17^ these outcomes may also be influenced by expanding and improving ease of access to healthcare for disengaged and under-insured populations, who are common among PWH. With renewed advocacy for Medicaid expansion in several states, it is important to understand how the ACA has changed coverage and, indeed, quality of care outcomes for marginalized groups, such as PWH.^3,9,18-21^

We therefore sought to use the quasi-experiment of staggered Medicaid expansion among some US states under the ACA to quantify the impact of Medicaid expansion on HIV care continuum outcomes compared to states that did not expand Medicaid. We evaluated this in the largest and most geographically diverse HIV cohort in the United States, the North American AIDS Cohort Collaboration on Research and Design (NA-ACCORD).^22^

## Methods

### Source population, study population, and follow-up

Individuals aged ≥18 years and in care in NA-ACCORD clinical cohorts between January 1, 2012 and December 31, 2017 were eligible for inclusion. Focusing on this study period allowed us to focus on short-term effects and examine the period when most states that went on to initiate Medicaid expansion actually expanded Medicaid.

The NA-ACCORD consists of single and multi-site clinical and interval cohort studies that accumulate data from adults with HIV at ≥200 sites in the United States and Canada; the NA-ACCORD consortium has been described in detail previously.^22^ Briefly, NA-ACCORD clinical sites collect standardized data on demographic and clinical characteristics (eg, participant age, birth sex, current gender, race/ethnicity, HIV acquisition risk category, state and ZIP code or province, and postal code of residence), antiretroviral medications (dates of regimen initiations and switches; protease inhibitor, non-nucleoside reverse transcriptase inhibitor, integrase strand transfer inhibitor, or other core class constituents; nucleoside/nucleotide reverse transcriptase inhibitor backbone constituents; etc.), laboratory values (eg, CD4+ count, CD4+ percent, and HIV-1 RNA viral load), clinical diagnoses (International Classification of Diseases, Ninth and Tenth Revisions (ICD-9 and ICD-10) diagnoses including AIDS-defining events, adjudicated myocardial infarction events, validated non-AIDS-defining cancers, etc.), and vital status including date of death on cohort participants as part of routine clinical care. Data are transmitted at regular intervals to a centralized Data Management Core at the University of Washington (Seattle, WA) for quality control and data harmonization across cohorts, after which they are transmitted to the Epidemiology/Biostatistics Core at Johns Hopkins University (Baltimore, MD). Institutional review boards at each participating site and at the Johns Hopkins University School of Medicine have reviewed and approved the activities of NA-ACCORD.

Individuals in all 50 US states and the District of Columbia contributed and were followed from enrollment in the NA-ACCORD until their final clinic visit before death or the end of the study period and censored thereafter.

### Exposure

State decisions to expand Medicaid under the ACA were assessed by calendar date, and exposure was attributed to all individuals living within the respective states in each calendar period.^23^ The 5 states (Alaska, Indiana, Louisiana, Michigan, and New Hampshire) that expanded Medicaid after January 1st were assumed to have expanded at the beginning of the year.

### Outcomes

Three HIV care continuum outcomes were measured: retention in clinical care, ART receipt, and viral suppression. An additional proxy measure of late HIV diagnosis and presentation to care, CD4 at enrollment, was also assessed.

Retention was defined with the National Academy of Medicine/National HIV/AIDS Strategy indicator as at least 2 HIV primary care visits within a calendar year separated by >90 days.^16,24^ Retention was assessed in each calendar year after the year of entry into the study.

Antiretroviral therapy receipt was defined using the US Department of Health and Human Services (DHHS) indicator: ART use at any time during the calendar year, among those with ≥1 clinic visit in that year.^24,25^ Antiretroviral therapy itself was defined as multidrug regimens including ≥3 antiretroviral agents, excluding triple-nucleoside regimens.^26^

Viral suppression was defined using the US DHHS indicator: HIV-1 RNA < 200 copies/mL at last measurement in the calendar year, among those with ≥1 clinic visit and HIV-1 RNA measurement in that year.^25,27^

CD4 at enrollment into care was defined as the first available CD4+ count within 6 months of first NA-ACCORD encounter date.^26,28,29^ Higher CD4 count at enrollment has long been used as a measure of reduced time between HIV infection and diagnosis and linkage to HIV care; we assessed it here as a marker of reducing delays in presentation to care and potentially a downstream proxy for improved access to HIV testing in expansion states.^28-30^

### Statistical analyses

We performed a modern difference-in-differences method to estimate the average treatment effect among the treated (ATT) for Medicaid expansion on the outcomes.^31^ This method used individual-level data from 2012-2017, accounting for staggered Medicaid expansion across calendar years.^31^ We used 2-group, pre-vs-post models for each outcome at each year, with non-expansion states as comparators and time-varying expansion status (exposure). The models also included region (Northeast, South, Midwest, or West based on US Census Bureau designation^32^), age at enrollment, race/ethnicity (Black non-Hispanic vs non-Black), and birth sex (male vs female). Age was included using a natural cubic spline with 3 knots in all models. The inclusion of these covariates was meant to balance or account for potential demographic differences in state populations that have also been shown in prior work to be associated with HIV care continuum outcome disparities.^33^ Though this does not imply that we conceptualize these factors as proper confounders (ie, they cannot be said to cause the exposure, namely statewide Medicaid expansion decisions), they are also associated with historical and social factors which have led to differences in the geographic distributions of socioeconomic and policy factors in the modern United States.^34-37^ The comparison group in each time period was the group of states that had not yet expanded by that period. The effect of Medicaid expansion on each outcome was the weighted average of average effects at each length of exposure during the post-expansion period; weights were proportional to the size of each exposure group. Due to data sparsity, CD4 models, which only included newly enrolled patients, only used data from 2013-2014 and adjusted for region as South vs other.

As sensitivity analyses to assess the robustness of inferences to differing model assumptions, we also fit comparative interrupted time series regression models using generalized estimating equations (GEE) with independent working correlation structures, propensity score-weighted models to estimate the average effect of Medicaid expansion in 2014 on the set of PWH who could be matched across expansion categories (propensity score logistic regression model included age, race/ethnicity, and sex, with all variables interacted),^30^ and an augmented synthetic control method using state-level summaries (ie, proportions or means) to estimate the impact of Medicaid expansion on our outcomes.^38,39^ We also assessed sensitivities of the primary analysis to measurement error in the exposure and to potential unmeasured confounding by other state-level characteristics. We did this first by excluding the following states from the primary analysis because they had more generous Medicaid income eligibility (>100% of the federal poverty level) prior to implementation of expansion under the ACA: Arizona, California, Delaware, the District of Columbia, Hawaii, New York, and Vermont. Second, we excluded 1 state at a time from the analysis to assess sensitivity of inferences for each outcome to potential state-specific factors that we could not account for in our primary analysis. Finally, we performed secondary analyses to assess the potential impact of Medicaid expansion on increased access to HIV care and general health services utilization, as found in prior work, by applying our difference-in-difference (DID) model to the annual changes in average new patient enrollment volume and in average total unique patient volume (including both new enrollees and re-engaging individuals) as additional outcomes.^19,40^

All analyses were executed in R version 4.0.3 (cran.r-project.org), and all scripts are archived at https://biostat.app.vumc.org/ArchivedAnalyses. Additional details are in Supplemental material.

## Results

**Figure 1.**
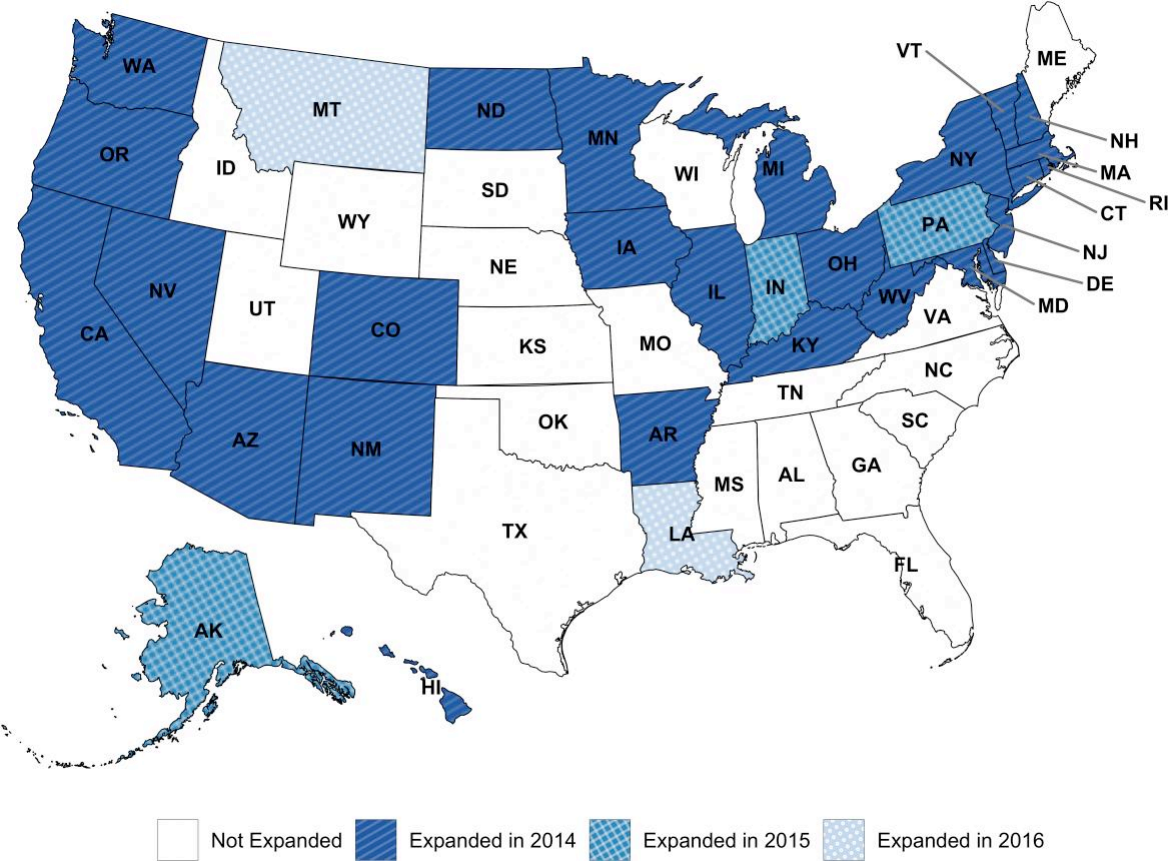
Medicaid expansion status, under the Patient Protection and Affordable Care Act (ACA), by US state, in 2014, 2015, and 2016.

In total, 26 states expanded Medicaid coverage under the ACA in 2014, 3 in 2015, and 2 in 2016 (Figure 1). Person-years of follow-up for demographic and outcome variables by expansion status and period (pre- vs post-expansion) are displayed in Table 1.

**Table 1.** Distribution of person-years across baseline characteristics among HIV + adults enrolled in care at North American AIDS Cohort Collaboration on Research and Design (NA-ACCORD) US sites from 2012-2017, by state status (Medicaid expanded and non-expanded) and time period (pre-expansion and post-expansion).

Characteristic	Total (person-years)	Expanded group during pre-expansion years	Non-expanded group during pre-expansion years	Expanded group during post-expansion years	Non-expanded group during post-expansion years
Age at baseline (years)	48 (30.66)	49 (33.65)	48 (31.65)	48 (31.65)	48 (30.66)
Sex					
Male	274 263 (84%)	163 079 (85%)	90 271 (83%)	170 614 (85%)	94 063 (83%)
Female	50 850 (16%)	28 187 (15%)	19 027 (17%)	29 259 (15%)	19 696 (17%)
Race/ethnicity					
White, non-Hispanic	120 870 (37%)	74 606 (39%)	38 051 (35%)	77 042 (39%)	39 701 (35%)
Black, non-Hispanic	135 096 (42%)	68 988 (36%)	56 442 (52%)	71 846 (36%)	58 661 (52%)
Hispanic	45 605 (14%)	30 263 (16%)	11 418 (10%)	31 812 (16%)	11 775 (10%)
Other/unk.	16 942 (5%)	11 364 (6%)	3365 (3%)	12 774 (6%)	3597 (3%)
HIV transmission risk factor					
MSM	122 940 (38%)	77 204 (40%)	32 703 (30%)	82 774 (41%)	34 975 (31%)
Hetero.	72 902 (22%)	38 991 (20%)	28 388 (26%)	40 482 (20%)	29 422 (26%)
PWID	45 135 (14%)	28 662 (15%)	14 721 (13%)	28 839 (14%)	14 749 (13%)
Other/unk.	90 302 (28%)	50 383 (26%)	35 094 (32%)	51 986 (26%)	36 222 (32%)

In a total of 325 113 person-years of follow-up, individuals were clinically retained 74% of the time, on ART during the vast majority (97%) of follow-up years, and virally suppressed in 85% of qualifying measurements; no viral suppression measures were available for 11% of follow-up years. The median CD4 at enrollment across all study years was 543 cells/mm^3^ (interquartile range 327, 718 cells/mm^3^).

The unadjusted proportions of PWH with clinical retention, ART use, and viral suppression from 2012 to 2017 are shown in Figure S1a-c by calendar year and their states’ Medicaid expansion status. Figure S1d similarly displays the observed mean CD4 at enrollment. The proportion of PWH clinically retained tended to be higher in expansion states before expansion and lower after expansion. The proportion using ART was high across both groups but tended to be higher in all years among PWH who did not reside in expansion states. In contrast, viral suppression tended to increase over the study period but was generally higher in expansion states. Similarly, CD4 at enrollment was higher in expansion states over the entire study period, suggesting earlier presentation to care.

**Figure 2.**
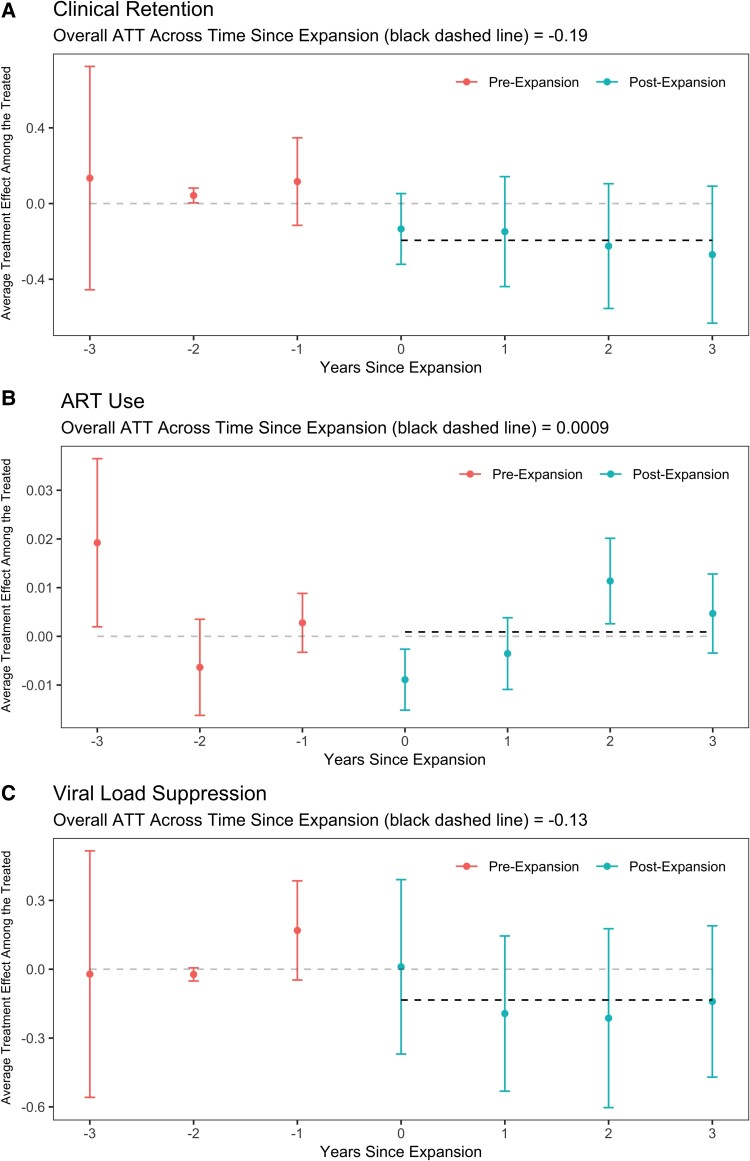
Estimates and 95% CIs of the differences in the proportion clinically retained (A), proportion receiving ART (B), and proportion virally suppressed (C) from difference-in-difference models assessing the association between Medicaid expansion status under the ACA, expansion period (as-yet unexpanded and currently expanded), stratified by length of time since expansion and adjusted for age, sex, race, and region. The parallel trends assumption conditional on these covariates can be partially tested with a test that ATT = 0 in the pre-expansion period; the *P*-values for this test were (A) *P* = 0.915, (B) *P* = 0.904, and (C) *P* = 0.179. ATT, average treatment effect among the treated.

Figure 2 shows the average treatment effects among the treated using the difference-in-differences method for retention, ART use, and viral suppression; these analyses were adjusted for age, sex, region, race/ethnicity, and geographical region. The overall average estimated effect of Medicaid expansion on retention was slightly negative (DID = −0.19 (95% CI: −0.38, −0.005). The estimated ATT for expansion on retention was fairly similar across years from expansion (−0.13, −0.15, −0.22, and −0.27 during years 0 [first year of expansion], 1, 2, and 3, respectively).The overall average effects of expansion on ART use (DID = 0.0009, 95% CI: −0.003, 0.005) and viral suppression (DID = −0.13, 95% CI: −0.36, 0.09) were not statistically different from zero. In the first year of expansion (year 0), ART use appeared to slightly decrease (DID = −0.009) whereas in year 2, ART use appeared to slightly increase (DID = 0.011). No such trends over time since expansion were seen for viral suppression. Using only 2013-2014 data to avoid data sparsity in initial CD4 counts, Medicaid expansion exhibited a significant positive effect on CD4 at enrollment (DID = 93.5, 95% CI: 52.9, 134 cells/mm^3^).

Among sensitivity analyses, adjusted comparative interrupted time series models with GEE using 2013-2014 data only indicated a slightly negative effect of Medicaid expansion on clinical retention in 2014 (Ratio of Odds Ratios [ROR] = 0.91, 95% CI: 0.86, 0.96) and a positive effect of Medicaid expansion on CD4 at enrollment in 2014 (DID = 32.8, 95% CI: 9.41, 56.15 cells/mm^3^ and DID = 38.8, 95% CI: 15.48, 62.26 cells/mm^3^) with region included either as Northeast, South, Midwest, or West or else as South or not South, respectively (Table S1; Figure S2). Propensity score methods (Table 2) indicated a negative effect of expansion in 2014 on clinical retention (OR = 0.86, 95% CI: 0.81, 0.90) and ART receipt (OR = 0.71, 95% CI: 0.61, 0.82) among the matchable set of patients, and a positive effect on viral suppression (OR = 1.20, 95% CI: 1.12, 1.28). A positive effect of expansion in 2014 on CD4 at enrollment was also observed (estimate = 68.2, 95% CI: 51.1, 85.3). In contrast, results of the augmented synthetic control method indicated no significant effects of Medicaid expansion on state-level proportions of PWH retained (estimate = 0.026, 95% CI: −0.06, 0.11), receiving ART (estimate = −0.005, 95% CI: −0.20, 0.01), or virally suppressed (estimate = 0.016, 95% CI: −0.03, 0.06). Mean CD4 at enrollment (estimate = −25.3, 95% CI: −70.0, 19.4 cells/mm^3^) also appeared unaffected. Pretreatment balance and estimated effects of Medicaid expansion averaged over state-level estimates for years since expansion are displayed in the Supplemental material (Figure S3).

**Table 2.** Estimates and 95% CIs from propensity score models assessing the association between Medicaid expansion status under the ACA, expansion period (pre- vs post-expansion), and HIV care continuum outcomes adjusted for region, age, sex, and race/ethnicity.

	Clinical retention	ART receipt	Suppressed HIV-1 RNA	CD4+ count at enrollment
	OR (95% CI)	OR (95% CI)	OR (95% CI)	B (95% CI)
Including region				
Expansion-by-period effect	0.96 (0.88, 1.05)	0.94 (0.73, 1.21)	1.11 (1.00, 1.23)	27.54 (10.72, 44.36)
Excluding region				
Expansion-by-period effect	0.86 (0.81, 0.90)	0.71 (0.61, 0.82)	1.20 (1.12, 1.28)	68.18 (51.10, 85.27)

Additional sensitivity analyses in which states with more generous pre-expansion Medicaid eligibility thresholds were excluded did not show appreciable differences from inferences in the primary analyses (Figure S4). These models showed minor negative effects that were non-significant for clinical retention and ART use (DID = −0.13 95% CI: −0.27, 0.006, and estimate = −0.004, 95% CI: −0.008, 0.001, respectively; Figure S4a and b), though the small negative effect was significant and sustained for viral suppression (DID = −0.22, 95% CI: −0.30, −0.14) due largely to later declines in the effect several years after expansion (Figure S4c). For the CD4 count at entry, however, as in the primary analyses, there remained a positive impact of Medicaid expansion, though somewhat smaller in magnitude (DID = 56.85 95% CI: 14.81, 98.90 cells/mm^3^). There were insufficient observations to adjust for region in this CD4 sensitivity analysis.

Similarly, sensitivity analyses in which one state at a time was excluded from the primary analyses did not show appreciable differences from the primary analyses across the exclusion of most states, excepting for the ART use outcome models excluding the state of Pennsylvania (in which the effect of Medicaid expansion on ART receipt probabilities was shifted in a positive direction away from the null; Figure S5b) and the mean CD4 outcome models excluding the state of Wisconsin (in which the effect of Medicaid expansion on changes in mean CD4 at entry moved toward the null; Figure S5d); the average effects with their resultant confidence intervals after the exclusion of each state are shown for each outcome in the Supplemental material (Figure S5).

Finally, secondary analyses assessing access to care through modeling new and total patient volume revealed a very small but non-significant increase in average new enrollees (estimate = 42, 95% CI: −139, 222; Figure S6a), and a small but non-significant decrease in average total patient engagement (estimate = −221, 95% CI: −710, 268; Figure S6b), in expansion vs non-expansion states in the post- vs pre-expansion periods.

## Discussion

In our study making use of a large, geographically, and demographically diverse population of clinically engaged PWH across the United States, we found a substantial positive effect of Medicaid expansion under the ACA on higher CD4 counts at the point of enrollment to care, though we failed to find clear and consistent evidence of a positive or negative effect on additional continuum of care outcomes downstream of successful linkage to HIV care. This is consistent with the overall positive impact of Medicaid expansion on HIV testing and linkage to HIV care that has been observed previously; it is also consistent with improved access to care for many other chronic conditions, and though the benefits were not as clear after successful engagement in HIV care, it does not contradict the benefits of robust publicly funded health programs targeted toward populations with significant health needs.

A major reason for the lack of an observed, consistent impact of Medicaid expansion on improving HIV continuum of care outcomes downstream of linkage to care could be the atypical HIV-specific safety net in the United States, the Ryan White HIV/AIDS Program (RWHAP). In theory, the ACA entailed several provisions to benefit individuals with chronic conditions, including those with HIV, among them more efficient treatment utilization, fewer emergency room visits, lower incidence of financial hardship such as uninsured hospitalizations,^4,7,41^ eliminating categorical eligibility, removing exclusions for “pre-existing conditions”, and reducing out-of-pocket drug costs due to the Medicare Part D “donut hole”.^42^ In spite of those beneficial provisions, the RWHAP has remained a critical and highly cost-effective source of support for PWH even since the implementation of the ACA and state Medicaid expansions.^8,42,43^ In fact, this reliance on RWHAP funding for services and medications in many southern states has been enhanced by those very same states’ refusal to expand Medicaid, leaving PWH residing therein fewer options to access affordable care.^42,44^ It is possible, then, that this robust safety-net program has obscured HIV outcome deficits that might otherwise be apparent in states foregoing Medicaid expansion. This interpretation aligns with prior analysis of the CDC's Medical Monitoring Project, which found viral suppression among those with Medicaid did not differ substantially from uninsured individuals, demonstrating how RWHAP “levels the playing field” for the uninsured.^45^

That said, the fact that HIV outcomes, particularly viral suppression and CD4 count at first encounter (an important marker of earlier engagement in care), may have been better in expansion than non-expansion states both pre- and post-expansion, and that proportions achieving successful outcomes, particularly ART receipt, neared 100% among some populations may have further limited our ability to observe sustained improvements after vs before Medicaid expansion due to “ceiling effects”.^46^ Though analyses examining newly enrolled groups of PWH have found both improvements and declines in viral suppression following Medicaid expansion, particularly among AIDS Drug Assistance Program (ADAP) clients, we did not observe those larger associations in our much broader population.^47-50^ Additionally, prior work that was able to assess HIV testing, the critical pre-diagnosis and pre-linkage portion of the care continuum, using a nationally representative survey from the Behavior Risk Factor Surveillance System, found increased test rates in Medicaid expansion states (+3.22 percentage points among those with incomes < 138% of the federal poverty level, compared to the same group in non-expansion states, in one study), with the largest impacts among key populations of younger, rural, and non-Hispanic Black males.^19,20^ However, one of those studies also found that HIV testing did not increase among Black and Hispanic females in expansion states, even though baseline HIV diagnosis rates were higher in those groups.^20^ Though we were unable to directly assess potential effects on the pre-linkage portion of the care continuum as Gai and Marthinsen or Menon et al. were, our examination of CD4 counts at enrollment did indicate a substantial positive impact on a commonly used proxy measure for improved HIV testing, timely HIV diagnosis, and swifter linkage to care.

Given that the population in our own study was already successfully linked to and engaged in care within the NA-ACCORD, however, we may have been less likely to observe Medicaid expansion effects on care continuum outcomes which are downstream of linkage (ie, retention in care, ART receipt, and viral suppression). This may be because Medicaid expansion would be expected to primarily benefit those unable to access care prior to its implementation. Additionally, we did not observe consistent negative effects of Medicaid expansion on ART receipt or viral suppression, in contrast with at least one other study which found poorer viral suppression among publicly insured individuals after Medicaid expansion, though this was solely in the District of Columbia.^51^

There may also be a dilution of observable effects for programs that primarily improve access to care when measuring those effects in a clinical cohort or network such as ours (as opposed to in a population-based or differently assembled cohort). For example, in the Women's Interagency HIV Study (WIHS), an interval cohort including both HIV-negative and HIV-positive women, a relatively recent analysis found that residence in a Medicaid expansion state was associated with a much lower risk of insurance coverage loss (relative risk of 0.62) and a higher probability of coverage gain (relative risk of 2.32) among all women.^52^ However, there are serious limitations to measuring clinical care engagement milestones in *non*-clinical cohorts.

Our findings of disparate but largely null effects among care continuum outcomes downstream of linkage could also be explained in part by our model assumptions and target estimands. For example, while our primary analysis estimated the average treatment effect among those exposed to Medicaid expansion, the comparative interrupted time series regression estimated the average treatment effect of Medicaid expansion among a clinically engaged population, and the propensity-score-matched models estimated the average treatment effect among the matched population in 2014, all at the individual level. The synthetic control analysis estimated yet a different average treatment effect, this time at the population (state) level. These different causal effects imply subtly different target populations (see Table 2 and Table S1 in Supplemental material).^31^

Among the multiple outcomes, Medicaid expansion did appear to have somewhat different effects in primary vs sensitivity analyses for retention in care, ART use, and viral suppression. We did see a significant slightly negative effect of Medicaid expansion under the ACA on clinical retention using both the DID and GEE regression approaches that assessed the average treatment effect in 2014. The slightly negative effects are still consistent with expectations set forth by Eaton and Mugavero,^53^ who noted that, even though increased insurance coverage was likely to be associated with improved engagement in care, specifically among low-income populations, it is possible that more engaged populations would be more likely to access alternative insurance options as well. This is also reflected in qualitative work that has found persistent difficulties in navigating Medicaid enrollment processes, among those newly enrolled, despite positive perceptions of Medicaid access after expansion.^21^ That said, altered engagement and retention in care due to Medicaid expansion efforts in expansion states may also presage a shifting clinical landscape during and following the COVID-19 pandemic, during which clinic visit modalities have, through both necessity and great ingenuity, been modified.^14^ It remains to be seen, however, how these modifications may necessitate changes in how we conceptualize clinical engagement and retention, as well as how we measure them. With respect to ART initiation and viral suppression outcomes, there have been few formal analyses of similar effects in prior work.^42,49^ Among the limited evidence to date, an analysis of ART use among 284 AIDS Drug Assistance Program (ADAP) recipients in Nebraska did show a 17% higher proportion of days covered (measured by the refill ratio) after Medicaid expansion in 2014, though this may have been more a function of an ADAP-specific policy of aggressively pursuing uninsured individuals for enrollment under the ACA in Nebraska, as opposed to Medicaid expansion specifically.^54^ Similarly, a 3-state analysis of ACA Qualified Health Program enrollees in Nebraska, South Carolina, and Virginia in 2015 found that viral suppression improved slightly after enrollment, with a standardized risk difference of 5.0% between enrollees and those remaining in direct ADAPs.^49^

CD4 counts at presentation, by contrast, were significantly higher in expansion vs non-expansion states, after vs before expansion, in both our primary and several of our sensitivity analyses. Similarly, studies in the pre-ACA era after state-specific insurance expansions occurred (eg, in Massachusetts) found that improved coverage was accompanied by increased CD4 counts at presentation, likely indicating the linkage of individuals who were diagnosed earlier in their disease course and who had lacked access to testing and care before expansion took place.^40,55^ This also was consistent with findings in our propensity-score-matched sensitivity analysis and further highlights that health insurance reform for PWH may need to focus on currently untested and unlinked individuals, as this group comprises a sizeable proportion (nearly 15%) of the HIV + population in the United States.^56^

Our analyses did, however, have multiple limitations. As noted above, our study population was drawn from clinical cohorts of PWH already successfully linked to and engaged in care, leaving our analyses vulnerable to limited transportability to groups who are yet undiagnosed or disengaged from care. Further, we could not account for the potential dilution of effects by RWHAP due to limited individual-level data on RWHAP-enrollment within the NA-ACCORD; as previously mentioned, there was a shift to Medicaid use in states that expanded, but in states that failed to expand, there was growth in RWHAP use for insurance coverage and treatment.^8^ In fact, almost 50% of PWH rely on RWHAP funding or services, with that percentage much higher (80%) among the uninsured.^9^ For our retention outcome, more recent clinical practice and potent ART regimens, including their attendant infrequent visit and laboratory testing requirements, may have rendered our kept-visit-based retention measure less reflective of patient engagement in care than in the past when these metrics were first formulated.^14^ These population-averaged effects may also obscure benefits accruing to some sub-populations in whom healthcare needs are more urgent; this focus on potential impacts of Medicaid expansion in key populations is the subject of complementary work by our colleagues (as yet unpublished). Finally, certain model assumptions necessary for valid inference in our primary analyses (eg, the conditional parallel trends assumption for difference-in-differences approaches) may have been violated.^39,57^ However, we did conduct multiple sensitivity analyses to provide a broader view of Medicaid expansion effects through different lenses and for subtly different estimands that were not subject to these same assumptions.

In this large cohort of PWH engaged in HIV care in the United States, we found positive impacts of Medicaid expansion on an indicator of more timely HIV diagnosis and linkage to care, namely higher CD4 at enrollment, but inconsistent effects for HIV care continuum outcomes that followed successful linkage and engagement. Our research adds both breadth and rigor to the current literature by leveraging the quasi-experiment of Medicaid expansion and employing multiple analytic approaches in such a large and diverse study population, which is generally representative of those receiving HIV care in the United States.^26^ Our work suggests that the RWHAP may also be a model safety-net program that more broadly available public insurance programs should emulate. Insofar as Medicaid expansion decisions remain fraught within several states that have so far refused it, articulating the likely benefits of expanded access to insurance for different populations remains a fruitful and necessary exercise.^18,44,58-61^

## Supplementary Material

qxae128_Supplementary_Data

## Data Availability

Data are available upon reasonable request and scientific review through the NA-ACCORD (see https://naaccord.org/).
