# Supplementary material for: The impact of Medicaid expansion under the Affordable Care Act on HIV care continuum outcomes across the United States
Source: Health Aff Sch. 2024 Oct 7;2(10):qxae128. doi: 10.1093/haschl/qxae128 (PMC11498052; doi:10.1093/haschl/qxae128)

**Supplemental Material**

**Figure S1**. Average unadjusted proportion of study population clinically retained (**a**), proportion receiving ART (**b**), proportion virally suppressed (**c**), and median CD4 count at entry (**d**) during the study period, stratified by Medicaid expansion status in the states in which they resided

(**a**)


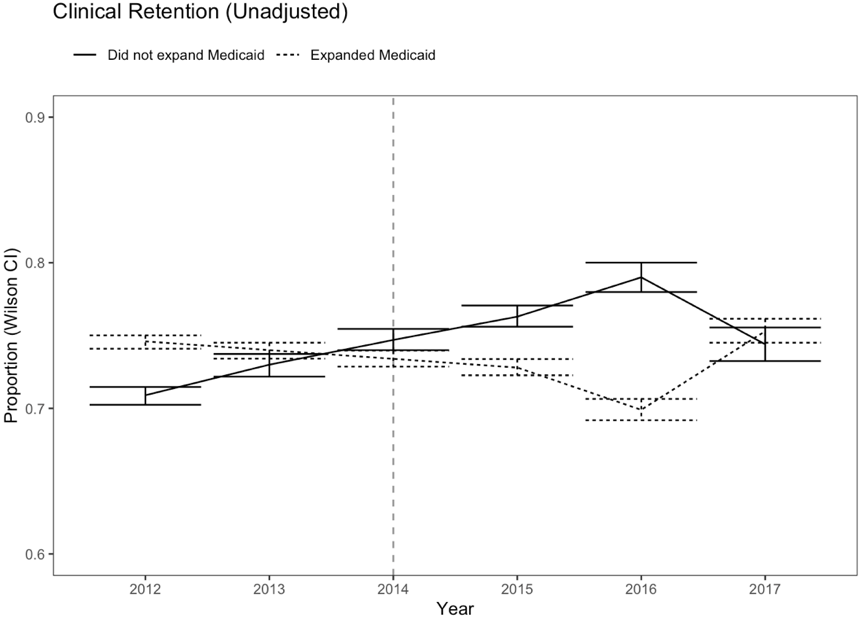


(**b**)


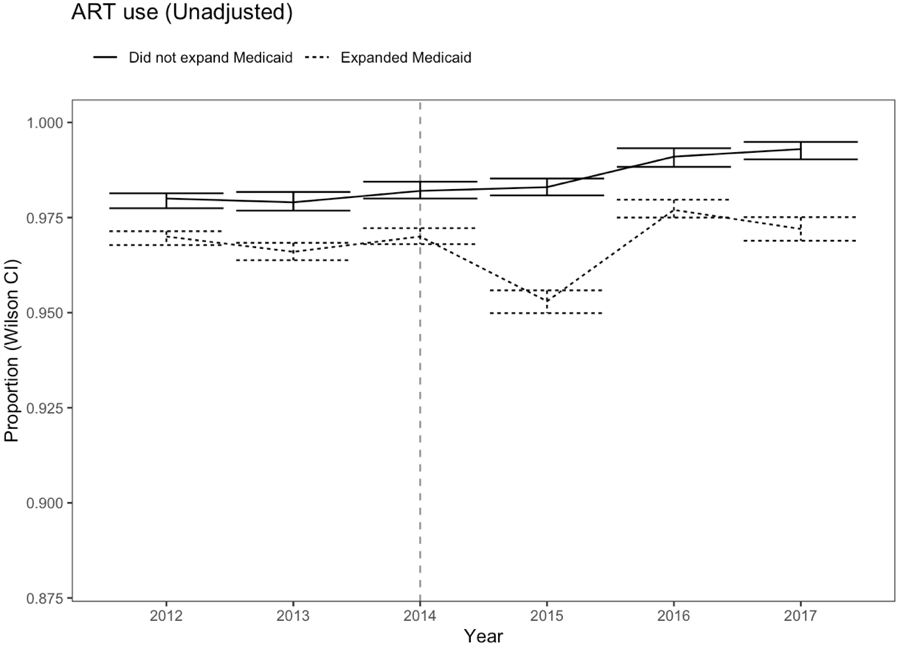


(**c**)


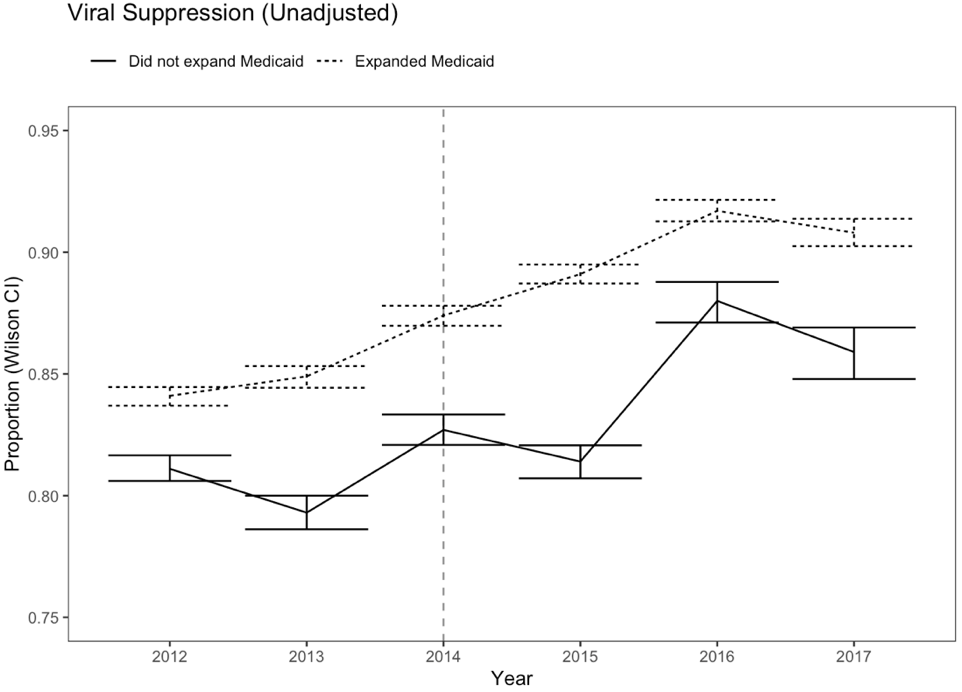


(**d**)


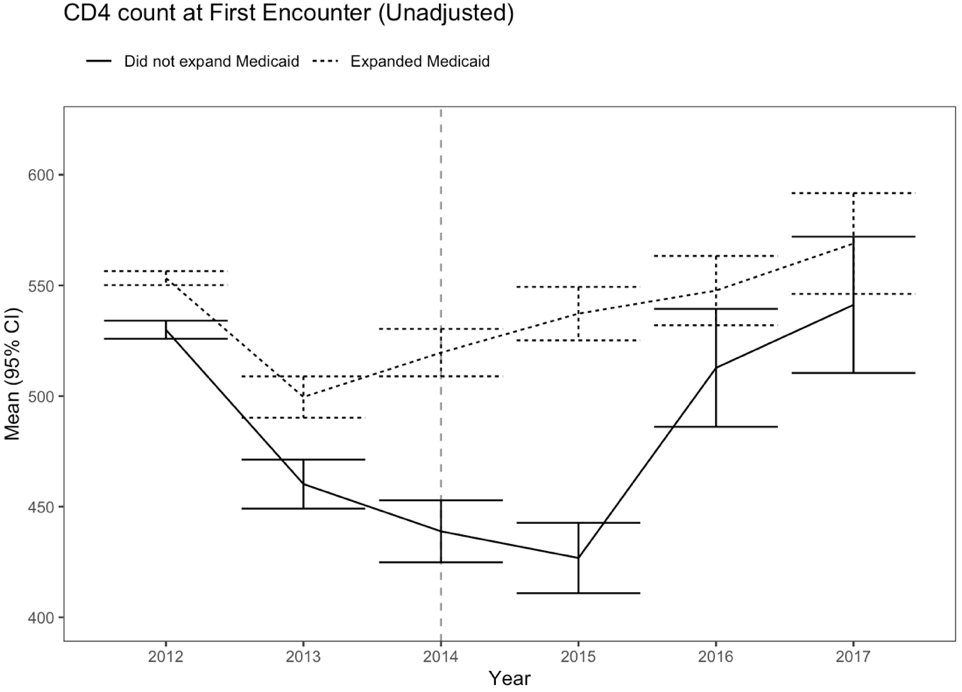


**Figure S2.** Expected probability of clinically retained (**a**), probability of receiving ART (**b**), probability of virally suppressed (**c**), and CD4 count at entry (**d**) during the study period, stratified by Medicaid expansion status in the states in which they resided, adjusted for age, race/ethnicity, and geographic region of the US (Northeast, South, Midwest, or West). Expected values calculated for a 48 year-old non-black male using GEE model fit with 2012-2017 data. Lines denoted as “Expanded in 2014” during the years 2012 and 2013 represent group that will eventually expand in 2014, but have not yet expanded.

(**a**)


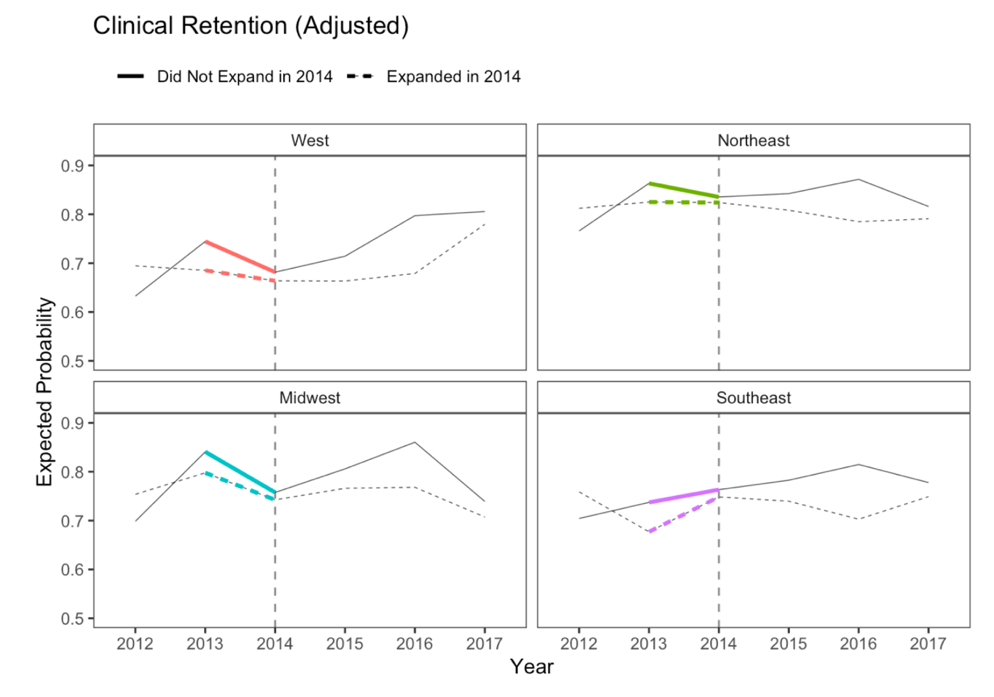


(**b**)


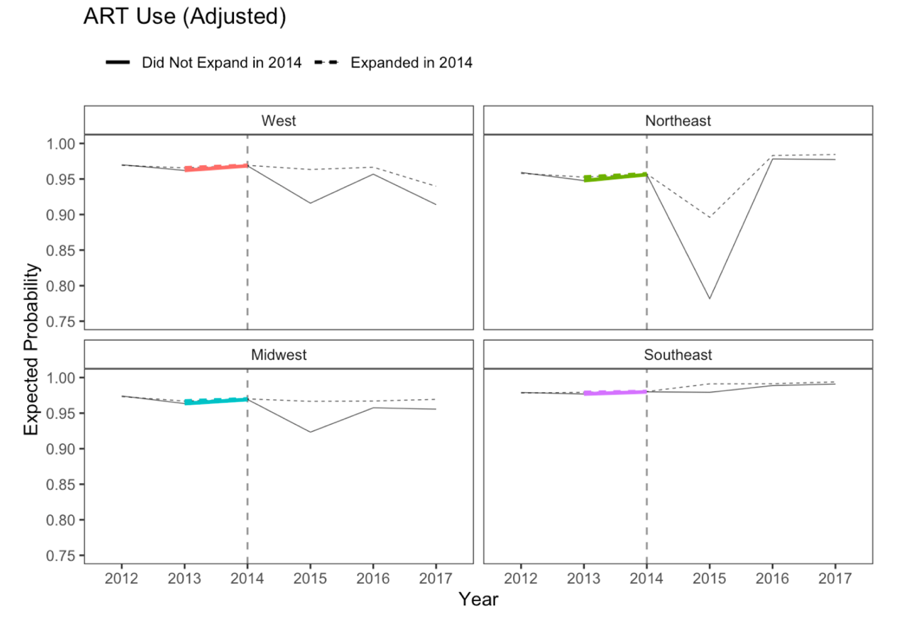


(**c**)


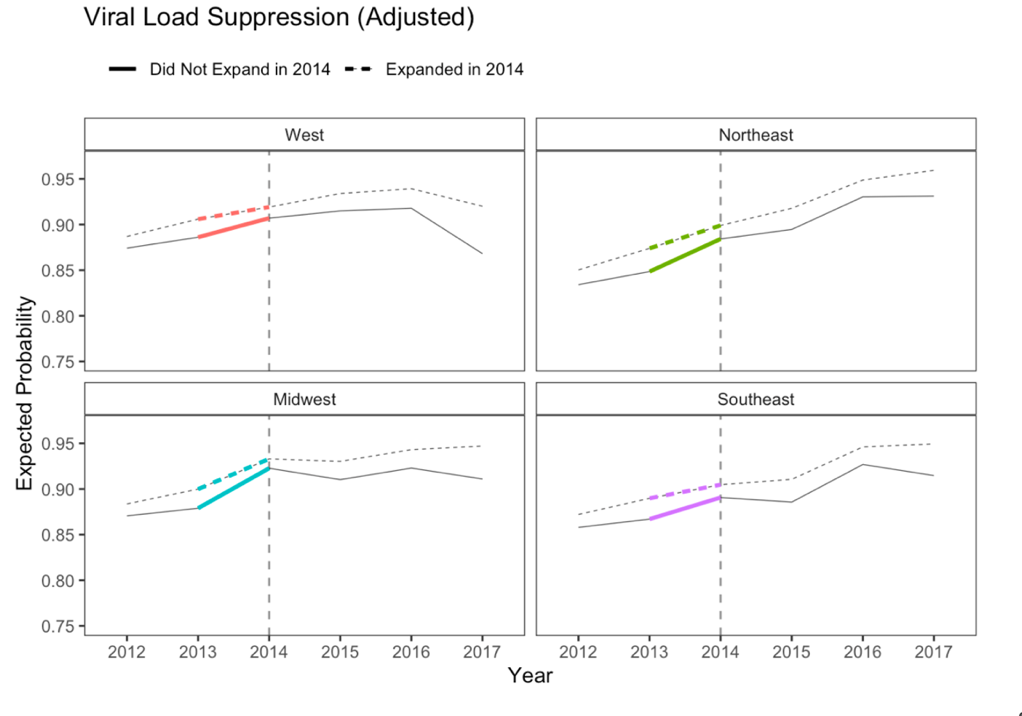


(**d**)


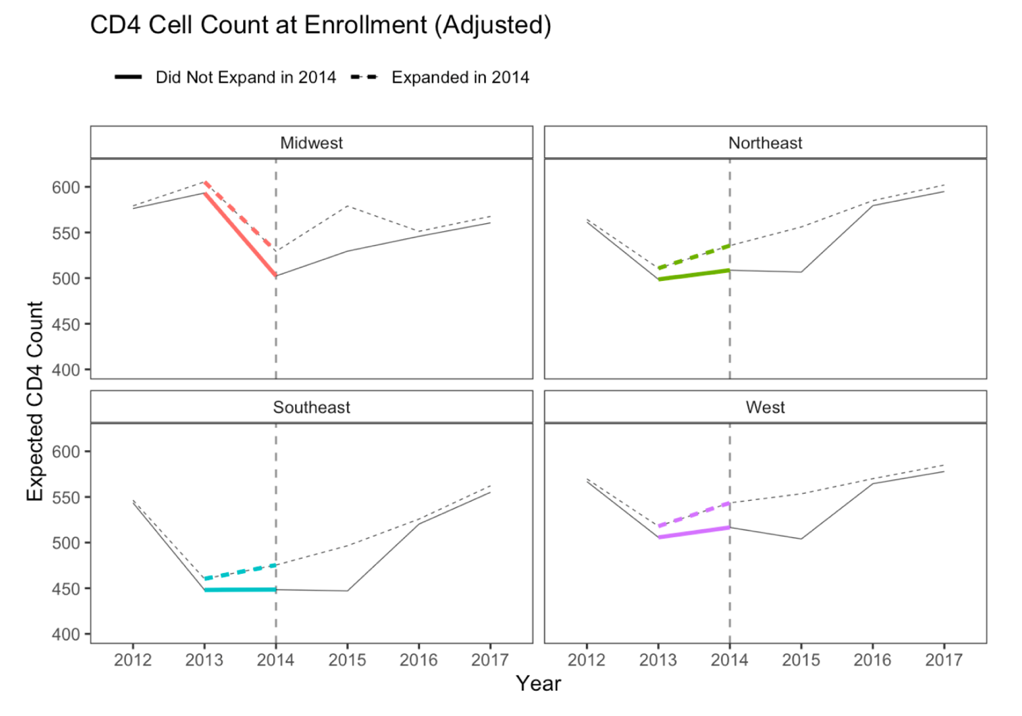


**Table S1.** Estimates and 95% confidence intervals (CIs) from regression models assessing the association between Medicaid expansion status under the ACA, expansion period (pre- vs. post-expansion), and HIV care continuum outcomes adjusted for region (Northeast, South, Midwest, or West), age, sex, and race/ethnicity. Results for CD4+ count at enrollment models are shown with region included in model as (a) Northeast, South, Midwest, or West and (b) South vs. Not South.

|  | **Clinical Retention** | **ART Receipt** | **Suppressed HIV-1 RNA** | **CD4+ Count at Enrollment** | |
| --- | --- | --- | --- | --- | --- |
|  | **ROR (95% CI)** | **ROR (95% CI)** | **ROR (95% CI)** | **(a) DID (95% CI)** | **(b) DID (95% CI)** |
|  |  |  |  |  |  |
| Expansion-by-Period Effect | 0.91 (0.86, 0.96) | 0.96 (0.86,1.05) | 0.97 (0.91, 1.04) | 32.8 (9.41, 56.15) | 38.8 (15.48, 62.16) |

**Figure S3.** Pre-treatment balance and average estimated effects of Medicaid expansion on the state-level proportion clinically retained (**a**), proportion receiving ART (**b**), proportion virally suppressed (**c**), and mean CD4 count at entry (**d**) using augmented synthetic control method. Nu (ν) is a hyperparameter that represents the ratio of the pooled fit across all states to the average state-level fit used as a weight to calculate the partially pooled fit.

(**a**)


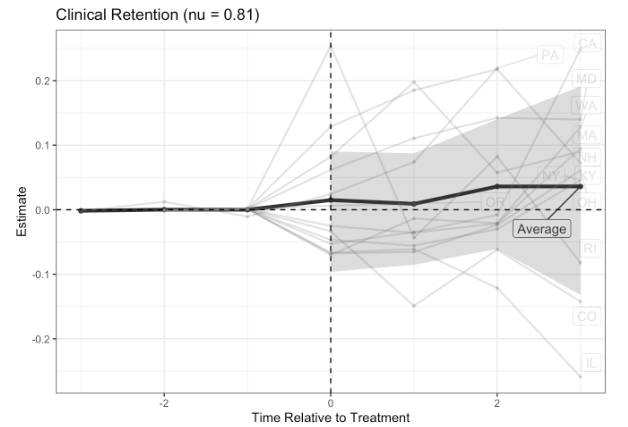


(**b**)


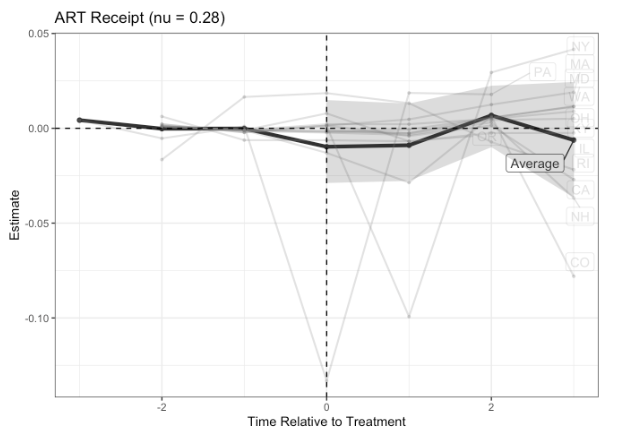


(**c**)


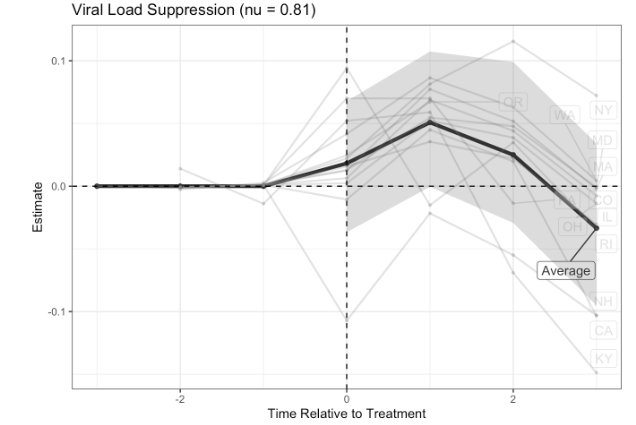


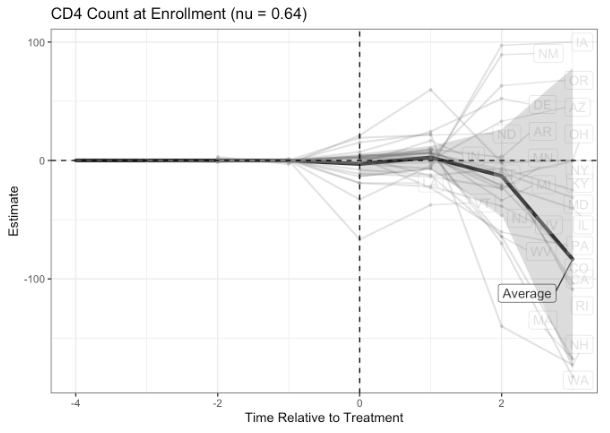
(**d**)

**Figure S4**. Estimates and 95% confidence intervals (CIs) of the differences in the proportion clinically retained (**a**), proportion receiving ART (**b**), and proportion virally suppressed (**c**) from Difference-in-Difference models assessing the association between Medicaid expansion status under the ACA, expansion period (as-yet unexpanded and currently expanded), stratified by length of time since expansion and adjusted for age, sex, race, and region, and excluding potentially misclassified states of Arizona, California, Delaware, the District of Columbia, Hawaii, New York, and Vermont (based on their more generous Medicaid eligibility requirements including income >100% of the federal poverty line in the pre-expansion period).


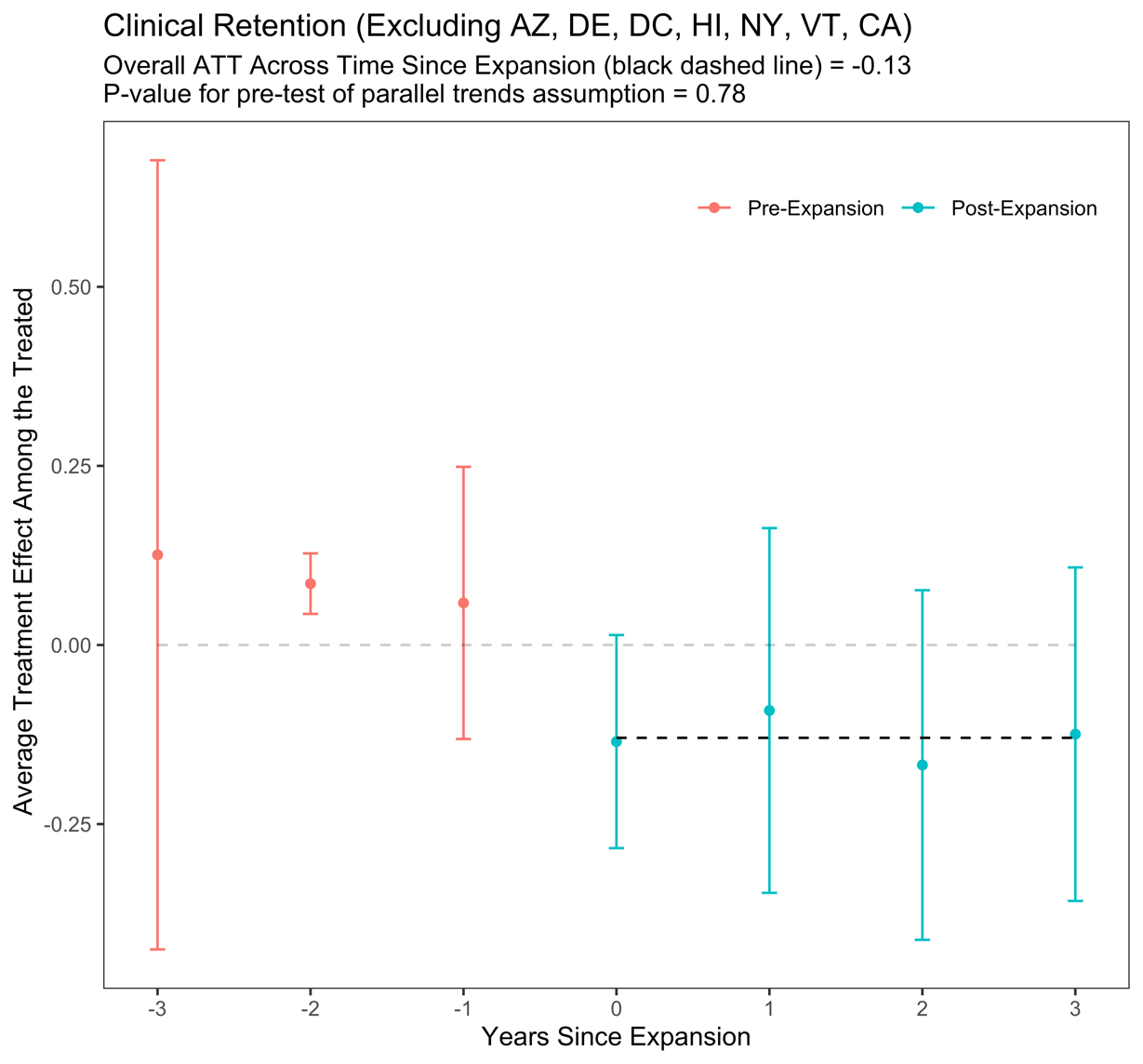


(**a**)


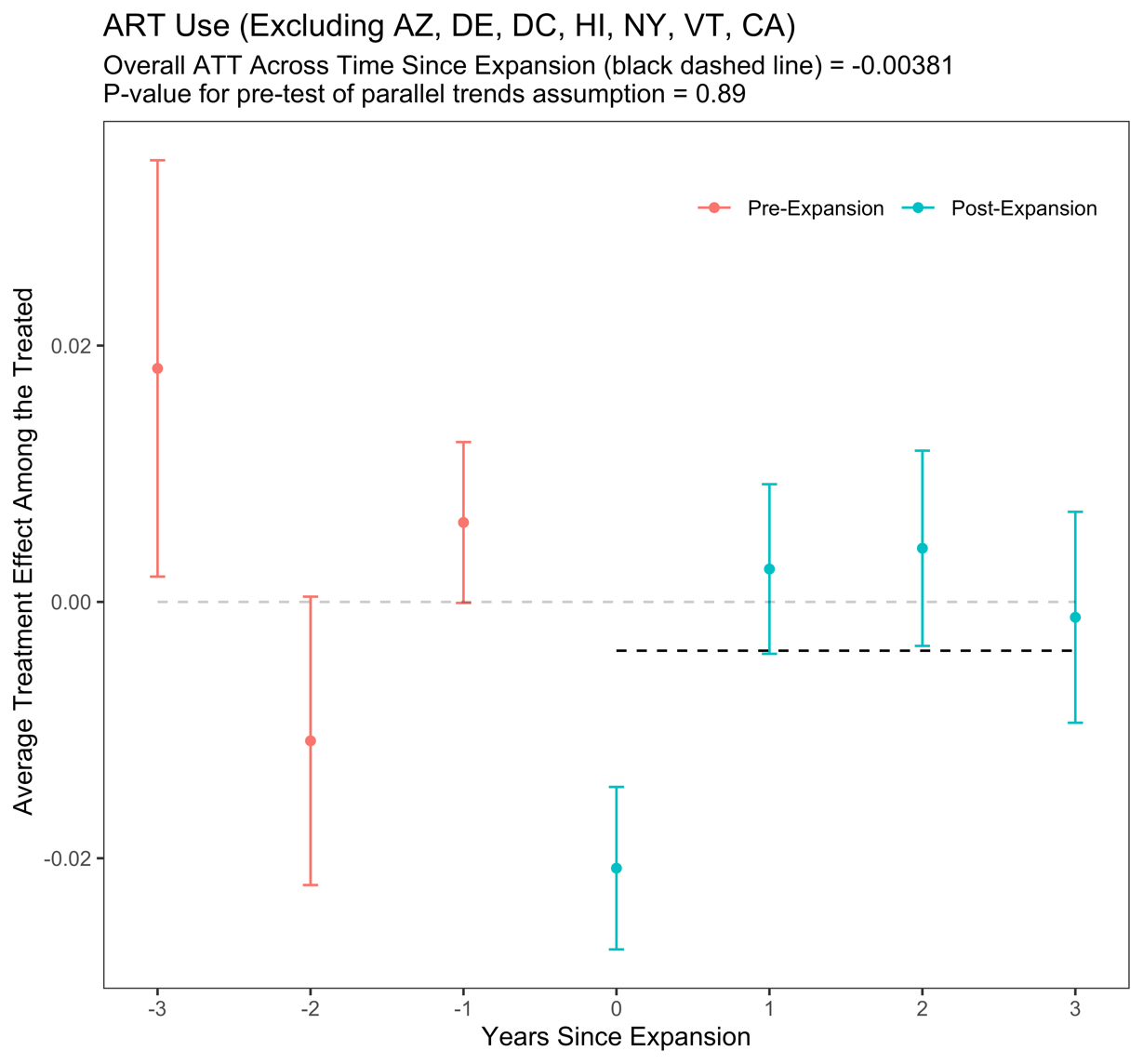
(**b**)


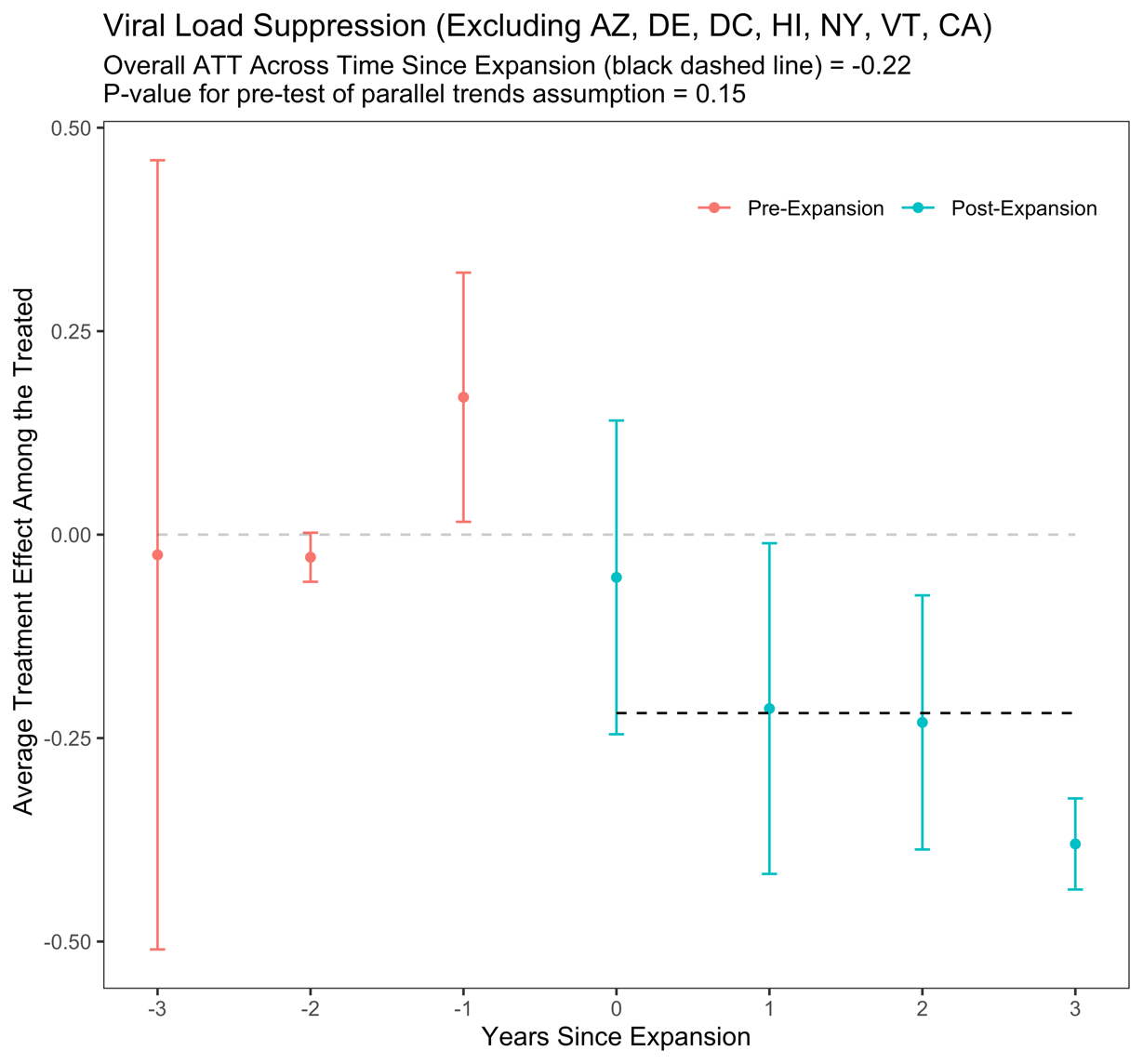
(**c**)

**Figure S5**. Estimates and 95% confidence intervals (CIs) of the differences in the proportion

clinically retained (a), proportion receiving ART (b), proportion virally suppressed (c), and CD4 count at entry (d), repeated in a jackknife sensitivity analysis excluding one state at a time from Difference-in-Difference models assessing the association between Medicaid expansion status

under the ACA, expansion period (as-yet unexpanded and currently expanded), stratified by

length of time since expansion and adjusted for age, sex, race, and region.

(**a**)


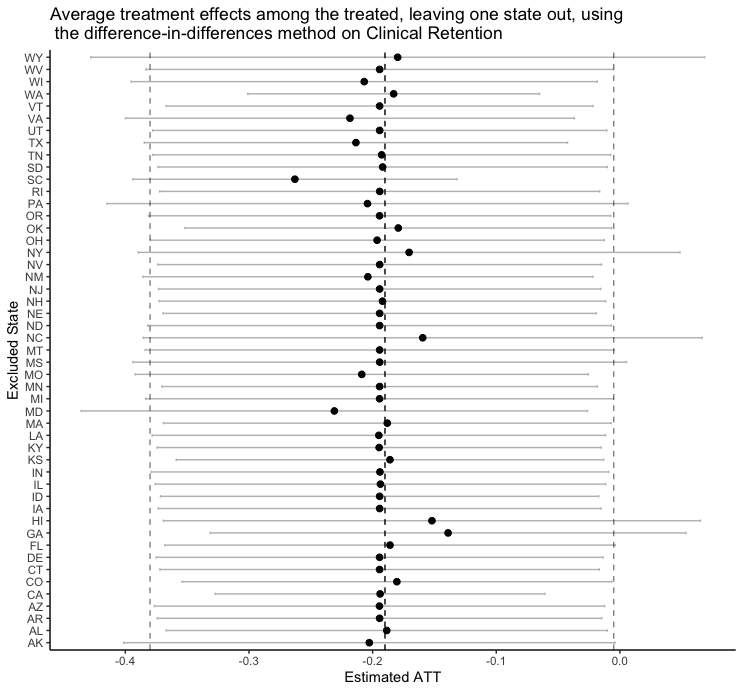


(**b**)


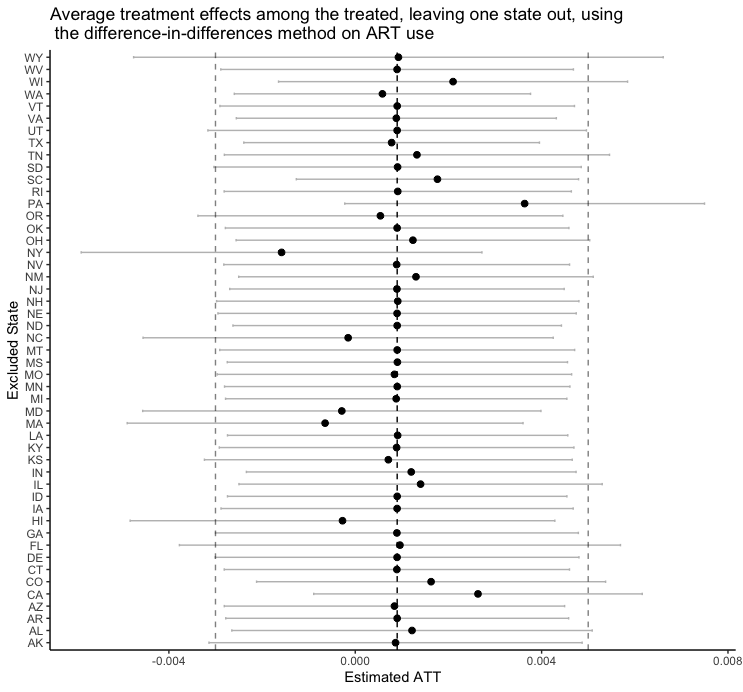


(**c**)


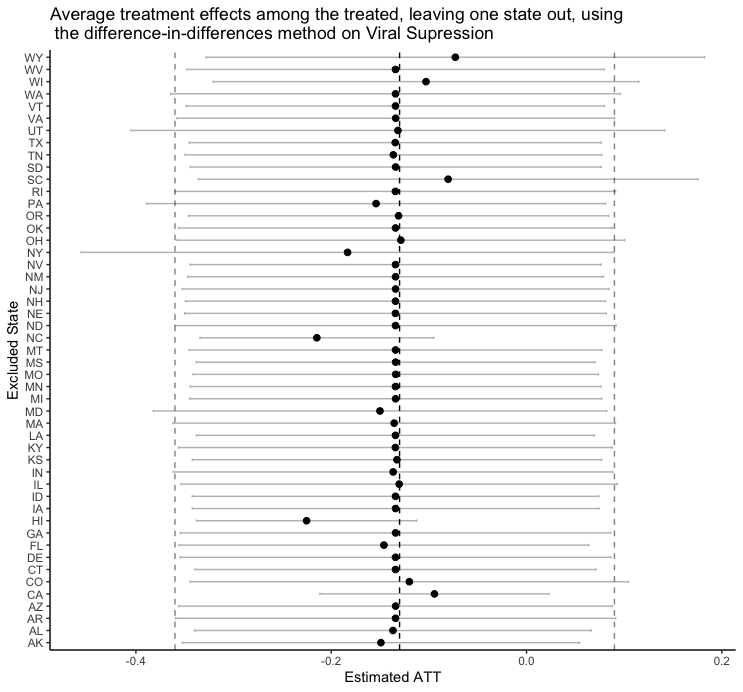


(**d**)


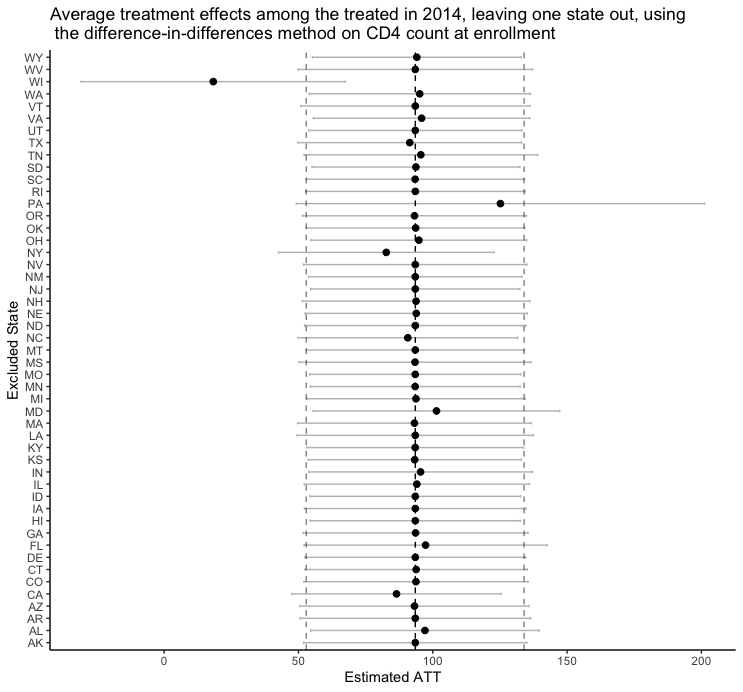


**Figure S6**. Estimates and 95% confidence intervals (CIs) of the differences in the average number of new enrollees in HIV care (**a**), and the total number of unique individuals receiving HIV care (whether new enrollees or those re-engaging in care) (**b**), in the NA-ACCORD from Difference-in-Difference models assessing the association between Medicaid expansion status under the ACA, expansion period (as-yet unexpanded and currently expanded), stratified by length of time since expansion and adjusted for age, sex, race, and region.


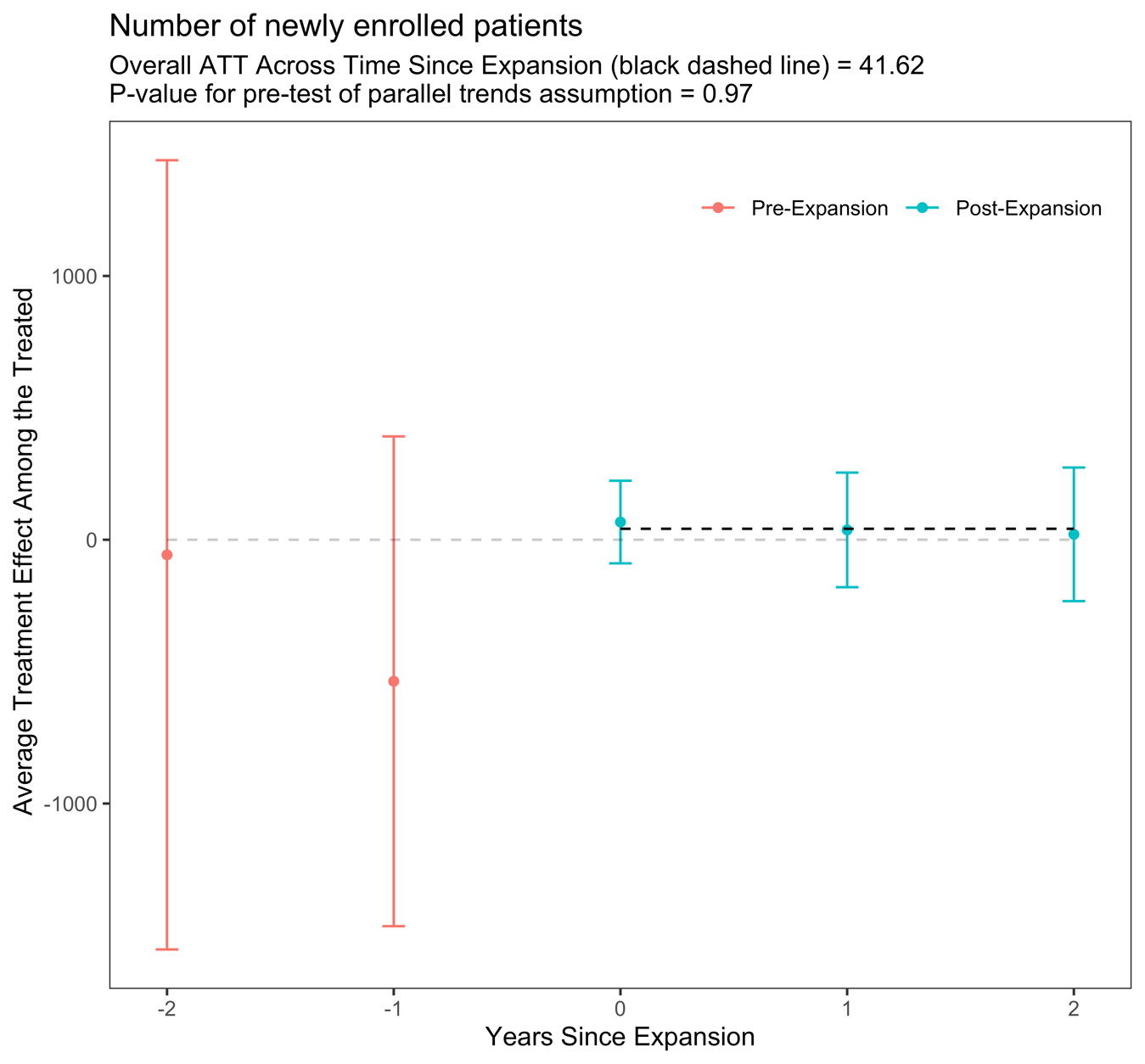
(**a**)


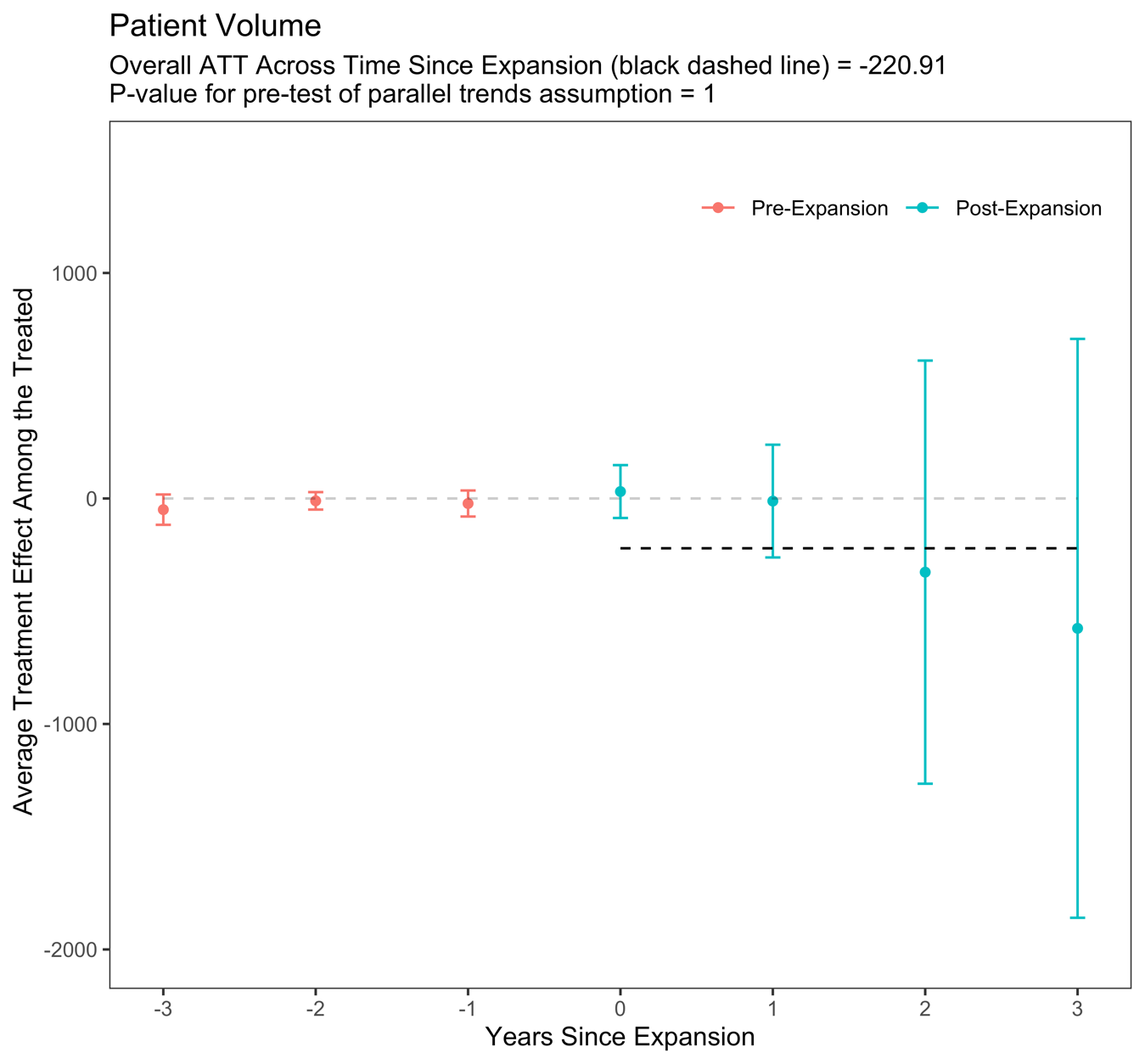
(**b**)


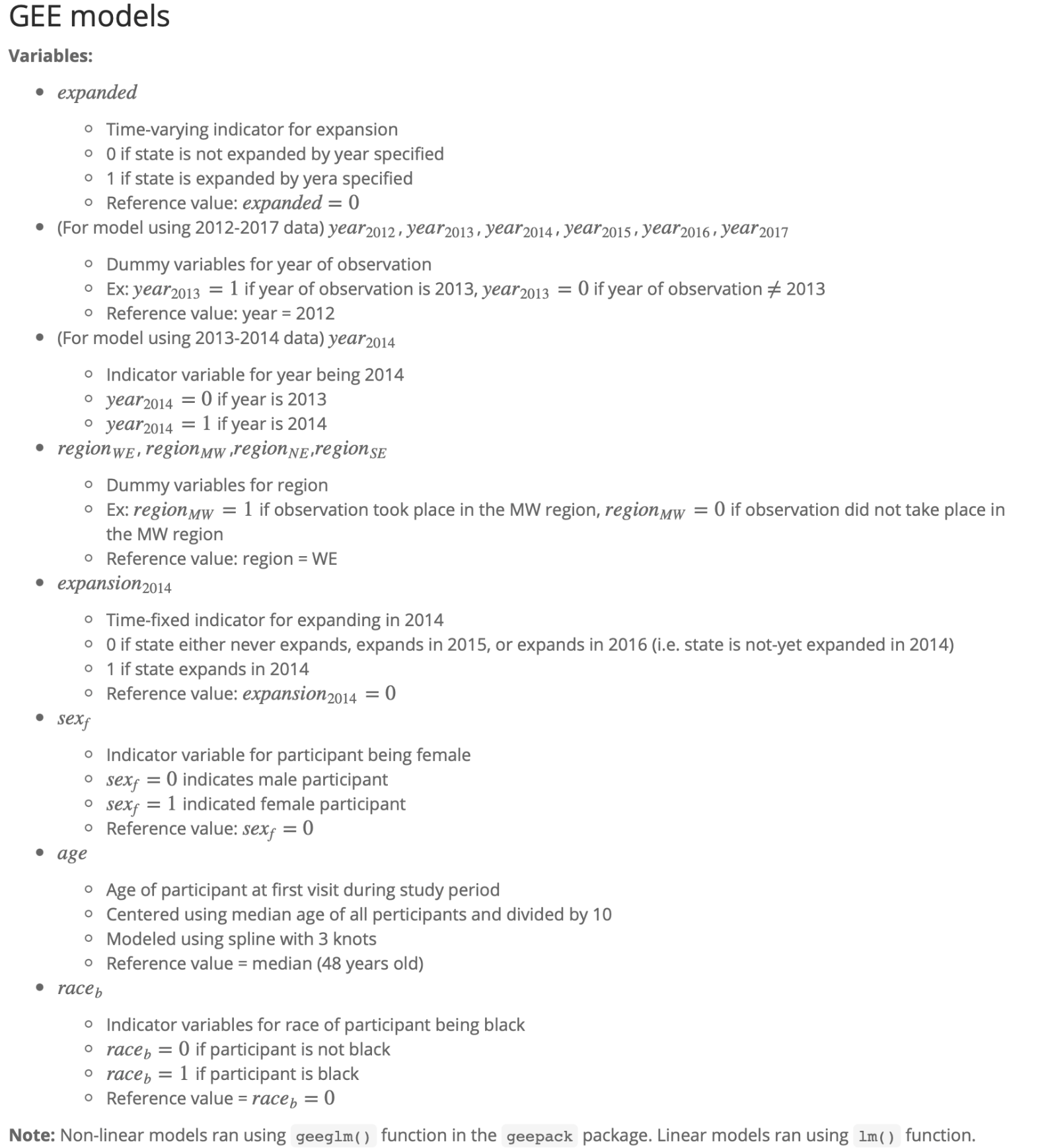


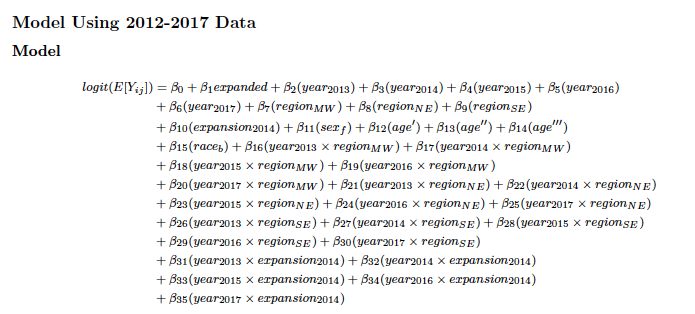


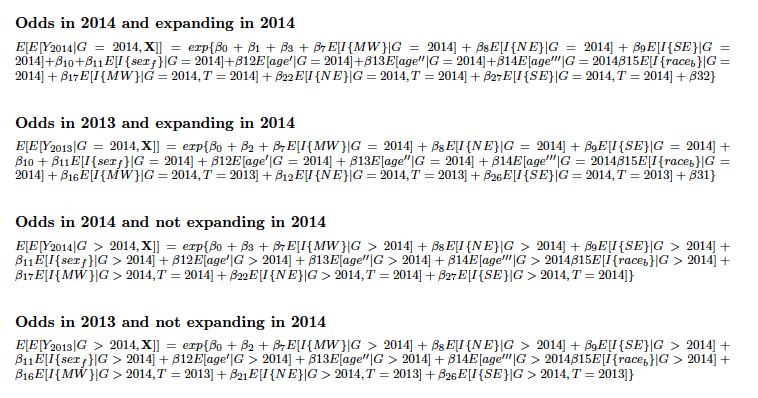


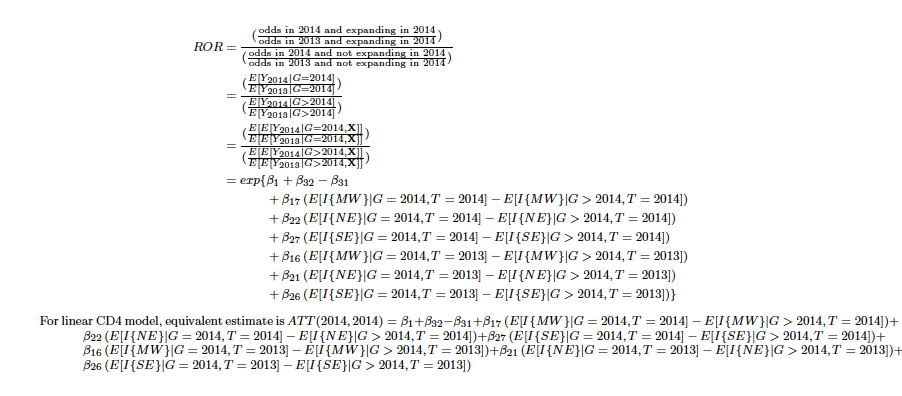


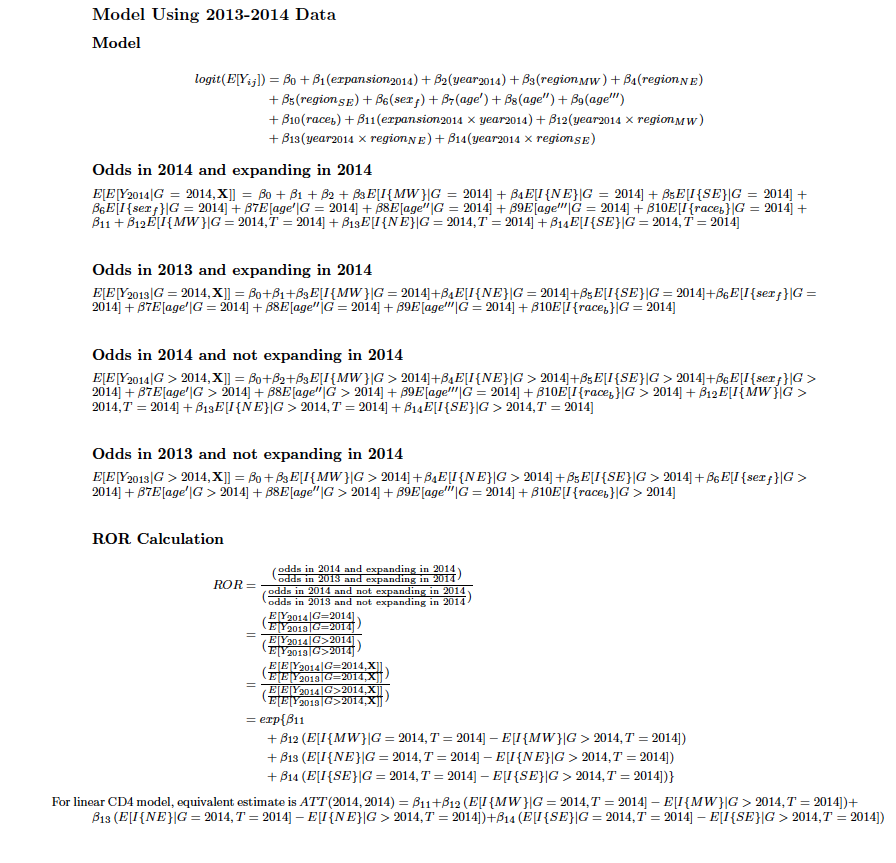

Supplement: qxae128_Supplementary_Data [file qxae128_supplementary_data.zip › N1501_ACA_Impact_HIV_CoC_Health_Affairs_SUPP_06Sep24.docx]
